# Immunogenicity of cell death and cancer immunotherapy with immune checkpoint inhibitors

**DOI:** 10.1038/s41423-024-01245-8

**Published:** 2024-12-10

**Authors:** Elena Catanzaro, Manuel Beltrán-Visiedo, Lorenzo Galluzzi, Dmitri V. Krysko

**Affiliations:** 1 Cell Death Investigation and Therapy (CDIT) Laboratory, Anatomy and Embryology Unit, Department of Human Structure and Repair, Faculty of Medicine and Health Sciences, Ghent University, Ghent, Belgium; 2 Cancer Research Institute Ghent, Ghent University, Ghent, Belgium; 3 Cancer Signaling and Microenvironment Program, Fox Chase Cancer Center, Philadelphia, PA USA

**Keywords:** Antigen-presenting cells, Chemotherapy, Clinical trials, CTLA4, Mouse models, Radiation therapy, PD-1, Targeted anticancer agents, Tumour immunology, Immune cell death, Immunotherapy

## Abstract

While immunotherapy with immune checkpoint inhibitors (ICIs) has revolutionized the clinical management of various malignancies, a large fraction of patients are refractory to ICIs employed as standalone therapeutics, necessitating the development of combinatorial treatment strategies. Immunogenic cell death (ICD) inducers have attracted considerable interest as combinatorial partners for ICIs, at least in part owing to their ability to initiate a tumor-targeting adaptive immune response. However, compared with either approach alone, combinatorial regimens involving ICD inducers and ICIs have not always shown superior clinical activity. Here, we discuss accumulating evidence on the therapeutic interactions between ICD inducers and immunotherapy with ICIs in oncological settings, identify key factors that may explain discrepancies between preclinical and clinical findings, and propose strategies that address existing challenges to increase the efficacy of these combinations in patients with cancer.

## Introduction

Immune checkpoint inhibitors (Box 1) have revolutionized the clinical management of multiple cancer types, including (but not limited to) melanoma [1], non-small cell lung carcinoma (NSCLC) [2], and head and neck squamous cell carcinoma (HNSCC) [3]. However, even in oncological settings in which ICIs are approved by the Food and Drug Administration (FDA) and other regulatory agencies worldwide, a large fraction of patients are refractory to immunotherapy with ICIs [4]. Moreover, ICI administration can be associated with nonnegligible short- and long-term toxicities [5]. Thus, substantial efforts have been dedicated to the identification of effective and safe combinatorial partners for ICIs for a variety of cancer types [6].

In this context, considerable attention has been given to the possibility of combining ICIs with standard-of-care (SOC) therapeutic regimens encompassing conventional chemotherapy, radiation therapy (RT) and/or targeted anticancer drugs, largely reflecting (1) the established safety profile of these agents [7, 8], (2) their ability to promote (at least some degree) tumor debulking [9], and (3) at least in some settings, their capacity to mediate therapeutically relevant immunostimulatory effects [10–12]. Indeed, one of the major determinants of resistance to ICIs in patients with cancer is scarce infiltration of the tumor microenvironment (TME) at baseline by cytotoxic T lymphocytes (CTLs) [13], which often correlates with (1) a reduced tumor mutational burden and hence a low neoantigen load [14] and (2) limited expression of the coinhibitory ligand CD274 (best known as PD-L1) [15].

**Fig. 1:**
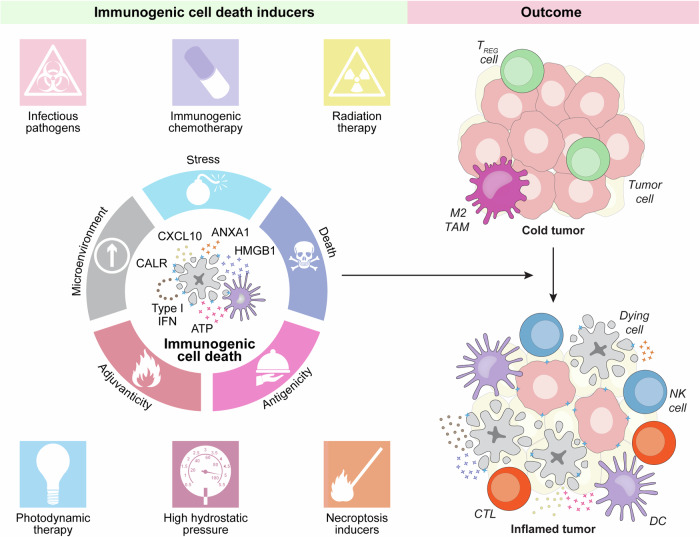
ICD as a tool for converting immunologically “cold” tumors into inflamed malignancies. Numerous biological, chemical and physical agents can elicit immunogenic cell death (ICD), a variant of regulated cell death that, in the context of failing to adapt to stress, antigenicity, adjuvanticity and permissive microenvironmental conditions, is sufficient to elicit adaptive immune responses specific for cell death-associated antigens that are associated with an active effector phase and the establishment of long-term immunological memory. Immunogenic cell death induction is generally associated with the abundant recruitment of immune effector cells and hence can (at least hypothetically) convert an immunologically cold tumor largely infiltrated by immunosuppressive M2-like tumor-associated macrophages (TAMs) and regulatory T (T_REG_) cells into an inflamed neoplasm exhibiting abundant infiltration by dendritic cells (DCs), cytotoxic T lymphocytes (CTLs) and natural killer (NK) cells. ANXA1 annexin A1, DAMP damage-associated molecular pattern, CALR calreticulin, CXCL10 C-X-C motif chemokine ligand 10, HMGB1, high mobility group box 1, IFN interferon

Several SOC therapeutics that can convert an immunologically “cold” (and hence ICI-insensitive) tumor into a “hot” neoplasm that exhibits an abundant CTL infiltrate and hence responds to ICIs (Fig. 1) belong to the class of immunogenic cell death (ICD, Box 2) inducers [16]. These therapeutics, which include specific chemical entities [10, 11, 17] as well as physical agents [18, 19], indeed, share the capacity to elicit a type of cell death that—in immunocompetent, syngeneic hosts—is sufficient to drive antigen-specific immune responses associated with an effector phase and the establishment of immunological memory [20].

While some ICD inducers have been shown to positively cooperate with ICI-based immunotherapy in the clinic, others have largely failed to unlock the full therapeutic potential of ICIs in patients with cancer [16]. Here, we critically discuss preclinical and clinical evidence of successful or unsuccessful interactions between ICD-inducing agents and immunotherapy with ICIs in oncological settings, with a specific focus on potential strategies to ameliorate the clinical efficacy of these combinations.

Box 1 Principles of cancer immunotherapy with ICIs Over the past 15 years, immune checkpoint inhibitors (ICIs) targeting cytotoxic T lymphocyte-associated protein 4 (CTLA4), programmed cell death 1 (PDCD1, best known as PD-1), CD274 (best known as PD-L1) or lymphocyte activating 3 (LAG3) have been included in the standard management of an increasing number of oncological indications, *de facto* revolutionizing cancer care [67]. From a mechanistic perspective, ICIs mostly operate by (re)activating tumor-targeting immune responses as mediated by CD8^+^ cytotoxic T lymphocytes and (at least in a fraction of malignancies) natural killer (NK) and γδ T cells [192–194]. In line with this notion, the likelihood of individual patients responding to ICIs (which significantly varies across tumor types) depends on (1) tumor infiltration by immune effector cells at baseline, which most often results in interferon gamma (IFNG)-dependent expression of PD-L1 by malignant and myeloid cells, and (2) tumor mutational burden, which is largely correlated with the ability of neoplastic cells to present antigenic determinants that are not covered by central tolerance and hence can effectively drive adaptive immunity [195]. Moreover, ICIs are generally associated with both acute and chronic immune-related adverse events (irEAs) that can affect a variety of organs, in a minority of patients with fatal complications [196, 197]. Currently, considerable efforts are being devoted not only to the characterization of other coinhibitory receptors that may be targeted by ICIs, such as hepatitis A virus cellular receptor 2 (HAVCR2, best known as TIM-3) and T-cell immunoreceptor with Ig and ITIM domains (TIGIT) but also (1) to the identification of predictive biomarkers other than PD-L1 expression and tumor mutational burden that may enable improved clinical decision making and (2) to the development of combinatorial therapeutic regimens that extend the clinical benefit afforded by ICIs in the context of manageable toxicity [195]. In this context, various immunogenic cell death (ICD)-inducing standard-of-care (SOC) treatments are being evaluated as combinatorial partners for ICIs in both preclinical and clinical settings (see main text).

Box 2 Principles of immunogenic cell death Immunogenic cell death (ICD) is a type of regulated cell death (RCD) that culminates with the activation of an antigen specific, adaptive immune response associated with an effector phase and the establishment of immunological memory, hence fundamentally differing from variants of RCD that are immunologically silent or elicit only inflammation [16]. Thus, ICD not only requires that dying cells express proteins encompassing antigenic determinants not covered by central tolerance but also requires (1) the spatiotemporally regulated emission of endogenous adjuvant-like signals that orchestrate the recruitment and activation of antigen-presenting cells (APCs) or the precursors thereof, and (2) the existence of microenvironmental conditions that are permissive for both the priming phase and the effector phase of immunity [198, 199]. Importantly, ICD can be executed via multiple RCD modalities, including apoptosis [200], necroptosis [201, 202] and (at least in some circumstances) ferroptosis [148, 203, 204]. Moreover, a number of biological, chemical and physical triggers can elicit ICD, such as viral pathogens, chemotherapeutic agents, radiation therapy (RT) and photodynamic therapy [16, 205]. Importantly, bona fide ICD is invariably associated with tumor infiltration by immune effector cells, notably dendritic cells (DCs), which are part of the priming phase, and CD8^+^ cytotoxic T lymphocytes (CTLs), which are part of the effector phase. Thus, while precisely determining whether a specific agent elicits ICD or other immunostimulatory effects that convert immunologically cold tumors into inflamed lesions remains challenging [137], ICD induction stands out as a promising strategy to improve the clinical efficacy of immune checkpoint inhibitors (ICIs) in patients with cancer (see main text).

## Successful interaction between ICD inducers and immunotherapy with ICIs

In a number of preclinical and clinical settings, chemical and physical agents eliciting ICD have been shown to positively interact with ICIs, resulting in superior therapeutic effects compared with either approach alone. Interestingly, in multiple (especially preclinical) scenarios, superior efficacy has been obtained by administering ICD inducers and ICIs according to specific, context-dependent schedules [21] and by using ICD-inducing chemotherapeutics according to metronomic doses [22, 23].

### Preclinical evidence

ICD-inducing conventional chemotherapeutics have been shown to cooperate with various ICIs in several syngeneic tumor models. For example, doxorubicin has been demonstrated to cooperate with ICIs specific for PD-L1, programmed cell death 1 (PDCD1, best known as PD-1), cytotoxic T lymphocyte-associated protein 4 (CTLA4) or CD96 in immunocompetent mice bearing subcutaneous 4T1 mammary carcinomas [24] or CT26 colorectal carcinomas (CRCs) [25, 26]. Similar results have been obtained by combining oxaliplatin with PD-1 or PD-L1 blockers in immunocompetent mice with subcutaneous MC38 or CT26 CRCs [26–28], PC3 prostate cancers [29], MOC1 HNSCCs [30], MB49 bladder tumors [31], H22 hepatocellular carcinomas [32], and MCA205 fibrosarcomas [33]. Notably, at least in some settings, such a therapeutic cooperativity could be further enhanced by immunostimulatory interventions beyond ICIs, including a Toll-like receptor 7 (TLR7) agonist [26, 34] and the systemic induction of autophagy with so-called caloric restriction mimetics (CRMs) [33, 35]. Similarly, a FOLFOX-mimicking chemotherapeutic regimen (which, among other components, includes oxaliplatin and 5-fluorouracil) reportedly synergizes with PD-1 blockers against CT26 CRCs developing in syngeneic immunocompetent BALB/c mice, a beneficial interaction that can be increased upon the codelivery of a monoclonal antibody specific for transforming growth factor beta 1 (TGFB1) [36].

Interestingly, while the ability of cisplatin to elicit bona fide ICD remains a subject of debate [37, 38], especially in clinical settings [39], this platinum derivative has been reported to synergize with a PD-1 blocker in immunocompetent mice bearing subcutaneous MOC1 HNSCCs [30], as well as with dual PD-1 and CTLA4 blockade in mice with established AB1 or AE17 mesotheliomas, resembling 5-fluorouracil [40]. The actual capacity of 5-fluororacil to cause ICD, however, is also controversial [41]. That said, both cisplatin and 5-fluorouracil have been shown to mediate therapeutically relevant immunostimulatory effects that may offer mechanistic ground for a positive interaction with ICIs [10]. Notably, another platinum derivative, PT-112, is a *bone fide* ICD inducer and indeed has been shown to synergize with PD-1 as well as PD-L1 blockers against subcutaneous TS/A mammary carcinomas developing in syngeneic immunocompetent BALB/c mice [42], suggesting that the ICD-inducing potential of platinum compounds depends on specific molecular features of the coordination complex and its cellular effects rather than on the platinum ion itself [10].

Other ICD-inducing chemotherapeutics that have been shown to synergize with ICIs in preclinical tumor models include (but are not limited to): (1) cyclophosphamide, which reportedly cooperates with PD-1 or CTLA4 blockers, optionally in the context of extra immunotherapeutic strategies such as adoptive cell transfer and vaccination, in mice bearing subcutaneous A20HA lymphomas [43] TC-1 lung carcinomas [44] or CT26 CRCs [45]; (2) mitoxantrone, which has been demonstrated to engage in positive therapeutic interaction with a PD-1 blockers in MCA205 fibrosarcoma-bearing mice, especially when combined with CRMs [33]; as well as (3) paclitaxel, which has been reported to cooperate with a PD-1 blocker against subcutaneous E0771 mammary carcinomas established in C57BL/6 mice [46].

For physical ICD inducers [47], RT delivered according to a hypofractionated schedule to a single neoplastic lesion has been demonstrated to synergize with CTLA4 and/or PD-1 blockers in immunocompetent BALB/c mice bearing bilateral TS/A mammary carcinomas or 344SQ lung carcinomas *s.c*., ultimately resulting in (at least some degree of) control of the contralateral, nonirradiated lesion [48–51], as well as in wild-type BALB/c mice with subcutaneous 4T1 mammary carcinomas, culminating with partial control of metastatic lung nodules [52]. In the 4T1 model, similar results have been obtained by combining RT with an ICI specific for V-set immunoregulatory receptor (VSIR, best known as VISTA) and metronomic cyclophosphamide [53]. Moreover, superior therapeutic benefits have been documented when RT was delivered in the context of CTLA4 or PD-1 blockade along with the following: (1) cyclophosphamide or doxorubicin, in immunocompetent C57BL/6 mice with subcutaneous mEER HNSCCs, B16 melanomas or MC38 CRCs [54, 55]; (2) the oncolytic peptide LTX-315 [56], in wild-type BALB/c mice bearing bilateral subcutaneous TS/A mammary carcinomas [57]; and (3) an agonistic antibody targeting TNF receptor superfamily member 9 (TNFRSF9, best known as CD137 or 4-1BB) or CD40 plus cyclophosphamide, in immunocompetent mice bearing AT3 mammary tumors *s.c*. or orthotopic ID8 ovarian carcinomas, respectively [58, 59]. Similarly, while photodynamic therapy has been shown to positively interact with PD-1 or PD-L1 blockers in various syngeneic mouse models of mammary carcinoma [60, 61] and CRC [60], low-dose pulsed ultrasound has been reported to synergize with a monoclonal antibody specific for PD-1 and doxorubicin in immunocompetent mice bearing orthotopic CT2A or GL261 glioblastomas [62].

Importantly, data from preclinical tumor models suggest that ICD inducers, as well as other (immuno)therapeutics, engage in superior interactions with ICIs when (1) the former are administered at low doses or according to metronomic schedules, rather than in an attempt to achieve a maximum-tolerated dose (MTD) [27, 32, 40], and/or (2) the former are administered according to specific schedules with respect to the latter, in a highly context-dependent manner [27, 31, 32, 63]. For example, mice bearing MC38 CRCs *s.c*. have been reported to respond to ICI-based immunotherapy only once previously administered a low dose (10 mg/kg) but not an ultralow (5 mg/kg) or high (20 mg/kg) dose of oxaliplatin, culminating in improved tumor infiltration by CD8^+^ CTLs, superior secretion of interferon gamma (IFNG), and consequently long-term disease control associated with the development of immunological memory [27]. Similarly, decreasing the total RT dose or RT dose per fraction has been associated with superior immunogenicity and hence synergistic interactions with ICIs in a variety of preclinical tumor models, including immunocompetent mice bearing TS/A mammary carcinomas [48, 50], ID8 ovarian carcinomas [59], and 344SQ lung adenocarcinomas [51]. Moreover, a single administration of low-dose oxaliplatin (3 mg/kg) has been shown to sensitize C57BL/6 mice bearing MC38 CRCs to ICIs targeting CTLA4 or PD-1 when delivered concurrently [64]. Similar results have been obtained with the concurrent (but not sequential) delivery of ICD-inducing chemotherapeutics or fractionated RT and an ICI targeting PD-L1 in preclinical models of CRC (CT26), triple-negative breast cancer (TNBC) (4T1) and glioblastoma [26, 65]. Conversely, the sequential (but not concurrent) administration of oxaliplatin upfront followed by a PD-1 blocker has been associated with superior therapeutic interactions in mice bearing MC38 CRCs [27]. Moreover, the delivery of cyclophosphamide before an ICI specific for CTLA4 has been demonstrated to eradicate CT26 CRCs established *s.c*. in immunocompetent BALB/c mice, an effect that was lost by the swapping administration schedule [45].

In summary, abundant preclinical data support the notion that ICD inducers can positively cooperate with ICIs in vivo, with a major, context-dependent impact for dose and administration schedule.

### Clinical evidence

A number of combinatorial therapeutic regimens involving one or more ICD inducer(s) (generally administered according to standard MTD approaches) and an ICI are currently approved by the FDA and other regulatory agencies worldwide for use in patients with cancer [66, 67], strongly supporting the notion that (at least in some oncological indications) the ability of cancer cells undergoing ICD to inflame the TME promotes ICI sensitivity (Table 1).

**Table 1:** Successful clinical trials testing ICD inducers and ICIs^a^

Indication	ICD inducer(s)	ICI(s)	Other	Trial Name	Trial #	Ref.
Breast cancer	Anthracycline, cyclophosphamide, paclitaxel	Nivolumab	Endocrine therapy, surgery	CheckMate 7FL	NCT04109066	[154]
Cervical cancer	Carboplatin, cisplatin, paclitaxel	Pembrolizumab	Bevacizumab	KEYNOTE-826	NCT03635567	[155]
Endometrial cancer	Doxorubicin, lenvatinib, paclitaxel	Pembrolizumab	n.a.	KEYNOTE-775	NCT03517449	[156]
Esophageal carcinoma	5-fluorouracil, cisplatin	Ipilimumab, nivolumab	n.a.	CheckMate 648	NCT03143153	[72]
Esophageal carcinoma	5-fluorouracil, cisplatin	Pembrolizumab	n.a.	KEYNOTE-590	NCT03189719	[157, 158]
Gastric carcinoma	Capecitabine, oxaliplatin	Sintilimab	n.a.	ORIENT-16	NCT03745170	[159]
Gastric or gastroesophageal junction adenocarcinoma	5-fluorouracil, capecitabine, cisplatin, oxaliplatin	Tislelizumab	n.a.	RATIONALE-305	NCT03777657	[160]
Gastric or gastroesophageal junction adenocarcinoma	5-fluorouracil, capecitabine, leucovorin, oxaliplatin	Ipilimumab, nivolumab	n.a.	CheckMate649	NCT02872116	[74, 161]
Gastric or gastroesophageal junction adenocarcinoma	5-fluorouracil, capecitabine, cisplatin, oxaliplatin, tegafur- gimeracil-oteracil potassium	Pembrolizumab	Trastuzumab	KEYNOTE-811	NCT03615326	[162]
Gastric or gastroesophageal junction adenocarcinoma	5-fluorouracil, capecitabine, cisplatin, oxaliplatin	Pembrolizumab	n.a.	KEYNOTE-859	NCT03675737	[73]
HNSCC	5-fluorouracil, carboplatin, cetuximab, cisplatin	Pembrolizumab	n.a.	KEYNOTE-048	NCT02358031	[163, 164]
HNSCC	Carboplatin, paclitaxel	Pembrolizumab	n.a.	KEYNOTE-B10	NCT04489888	[165]
NSCLC	Carboplatin, paclitaxel	Nivolumab	Bevacizumab	TASUKI-52	NCT03117049	[166, 167]
NSCLC	Carboplatin, paclitaxel	Atezolizumab	Bevacizumab	IMpower150	NCT02366143	[168, 169]
NSCLC	Carboplatin, cisplatin, pemetrexed	Pembrolizumab	Dexamethasone, folic acid, vitamin B_12_	KEYNOTE-189 MK-3475-189	NCT02578680 NCT03950674	[170, 171]
NSCLC	Carboplatin, nab-paclitaxel, pemetrexed	Atezolizumab	n.a.	IMpower130	NCT02367781	[172]
NSCLC	Carboplatin, cisplatin, paclitaxel, pemetrexed	Ipilimumab, nivolumab	n.a.	CheckMate 9LA	NCT03215706	[173, 174]
NSCLC	Carboplatin, cisplatin, nab-paclitaxel, paclitaxel, pemetrexed	Pembrolizumab	n.a.	MK-3475-A86	NCT04956692	n.a.
NSCLC	Cisplatin, etoposide, RT	Nivolumab	n.a.	RTOG 3502	NCT02768558	n.a.
NSCLC	Carboplatin, nab-paclitaxel, paclitaxel	Pembrolizumab	n.a.	KEYNOTE-407	NCT02775435 NCT03875092	[175–177]
SCLC	Carboplatin, cisplatin, etoposide	Pembrolizumab	n.a.	KEYNOTE-604	NCT03066778	[178, 179]
SCLC	Carboplatin, etoposide	Atezolizumab	n.a.	IMpower133	NCT02763579	[180–182]
TNBC	Cyclophosphamide, doxorubicin, nab-paclitaxel	Atezolizumab	Filgrastim	IMpassion031	NCT03197935	[183]
TNBC	Nab-paclitaxel	Atezolizumab	n.a.	IMpassion130	NCT02425891	[68]
TNBC	Carboplatin, gemcitabine, nab-paclitaxel	Pembrolizumab	n.a.	KEYNOTE-355	NCT02819518	[71]
TNBC	Carboplatin, cyclophosphamide, doxorubicin, epirubicin, paclitaxel	Pembrolizumab	Filgrastim	KEYNOTE-522	NCT03036488	[184]
TNBC	Nab-paclitaxel	Atezolizumab	n.a.	ANASTASE	NCT05609903	[69]

*HNSCC* head and neck squamous cell carcinoma, *ICD* immunogenic cell death inducer, *ICI* immune checkpoint inhibitor, *n.a.* not applicable, *NSCLC* non-small cell lung carcinoma, *RT* radiation therapy, *SCLC* small cell lung carcinoma, *TNBC* triple-negative breast cancer

^a^Limited to Phase II or higher; last updated: 10/17/2024

The IMpassion130 trial, in which patients with metastatic TNBC were randomly allocated to nab-paclitaxel plus placebo or atezolizumab (a PD-L1 blocker), demonstrated a significant overall survival (OS) advantage for individuals bearing PD-L1^+^ tumors in the combination arm (median OS: 25.0 *vs*. 15.5 months), an effect that was reduced in the intention-to-treat population [68]. Although exploratory, these data were largely confirmed in the ANASTASE phase III study [69], paving the way for the approval of this regimen as a first-line therapy for patients with PD-L1^+^ TNBC, even though the IMpassion131 trial failed to recapitulate the findings of IMpassion130 [70], which led the FDA to issue an official alert (https://www.fda.gov/drugs/resources-information-approved-drugs/fda-issues-alert-about-efficacy-and-potential-safety-concerns-atezolizumab-combination-paclitaxel#:~:text=On%20September%208%2C%202020%2C%20the,or%20metastatic%20triple%20negative%20breast). Multiple randomized, phase III clinical trials reported a progression-free survival (PFS) and/or an OS survival advantage for patients with TNBC receiving SOC chemotherapy plus pembrolizumab vs chemotherapy alone. For example, the KEYNOTE-355 trial documented both a PFS and an OS advantage in the PD-L1^+^ patient population (PFS: 9.7 months *vs* 5.6 months; OS: 23.0 months *vs* 16.1 months) [71], a beneficial effect that was less pronounced in the intention-to-treat population [71] and largely confirmed by the CheckMate 648 [72] and KEYNOTE-859 [73] trials. Similarly, the PD-1 blocker nivolumab combined with FOLFOX (folinic acid, 5-fluorouracil, and oxaliplatin) or XELOX (capecitabine and oxaliplatin) has been shown to provide superior PFS and OS advantages to previously untreated patients with HER2^+^ advanced gastric cancer, gastroesophageal junction cancer and esophageal adenocarcinoma enrolled in the CheckMate 649 trial, especially in subjects with a PD-L1 combined positive score ≥ 5, obtaining regulatory approval as first-line therapy for these oncological indications [74].

Alongside concurrent treatment schedules, therapeutic regimens combining an ICD inducer upfront followed by an ICI have also been shown to provide superior clinical benefits in various oncological settings. Thus, the phase II TONIC trial aimed to determine the best ICD inducer to condition the TME of patients with TNBC to maximize the clinical response to nivolumab [75]. Specifically, these patients were randomly allocated to receive hypofractionated RT in 3 fractions of 8 Gy each, cyclophosphamide (50 mg orally daily for 2 weeks), cisplatin (40 mg/m^2^ weekly for 2 weeks), or doxorubicin (15 mg weekly for 2 weeks), followed by SOC nivolumab administration. Across treatment arms, the overall response rate (ORR) was 20%, and even though cyclophosphamide and cisplatin followed by nivolumab were associated with a lower ORR than nivolumab monotherapy was, low-dose doxorubicin appeared to effectively sensitize TNBC to immune checkpoint inhibition (ORR: 35%), comparing well to nivolumab monotherapy (ORR: 17%) [75]. Similarly, the phase III PACIFIC trial documented a significant PFS (16.8 months *vs* 5.6 months) and OS (3-year OS: 57% *vs* 43.5%) advantage in patients with unresectable stage III NSCLC who did not progress on definitive, platinum-based, chemoradiation therapy subsequently receiving durvalumab (a PD-L1 blocker) *vs* placebo [76, 77]. While whether such an effect depends on a positive therapeutic interaction between chemoradiation and durvalumab rather than on the activity of the latter remains to be determined, these findings led to the approval of durvalumab as an SOC for patients with unresectable stage III NSCLC following chemoradiotherapy, marking a significant advancement in the treatment of this challenging patient population.

Recent clinical findings also support the use of neoadjuvant chemotherapy-immunotherapy combinations, especially durvalumab-based regimens, in patients with TNBC [78, 79]. Patients enrolled in the phase II GeparNuevo trial, which were randomly allocated to receive a single durvalumab injection prior to or along with chemotherapy with nab-paclitaxel, epirubicin, or cyclophosphamide followed by surgery, did not achieve a pathological complete response more frequently than patients receiving neoadjuvant chemotherapy only (53% *vs* 44%, not significant) [80]. However, compared with patients treated with neoadjuvant chemotherapy alone, individuals in the study arm that included durvalumab had significant benefits in terms of 3-year invasive disease-free survival (85.6% *vs* 77.2%), 3-year distant disease-free survival (91.7% *vs* 78.4%) and 3-year OS (95.2% *vs* 83.5%) [78, 80]. Interestingly, tumor-infiltrating lymphocytes were increased by treatment in both study arms [80], suggesting that while blocking PD-1 may activate a clinically relevant TNBC-targeting immune response in this patient population, ICD induction by chemotherapy may be required to enable abundant tumor infiltration by immune effector cells. Similarly, the open-label phase III CheckMate 816 trial, which compared neoadjuvant chemotherapy (either carboplatin plus paclitaxel, gemcitabine plus cisplatin, or pemetrexed plus cisplatin) combined with nivolumab to neoadjuvant chemotherapy only in patients with NSCLC, documented statistically significant benefits for ICI-containing regimens in terms of median event-free survival (31.6 months *vs* 20.8 months) and pathological complete response rates (24.0% *vs* 2.2%) [81]. Similar results have been obtained in the Phase III KEYNOTE-671 [82] and NADIMII [83] trials. Moreover, impressive results have also been achieved with neoadjuvant ICI-based immunotherapy alone in patients with melanoma [84–86], locally advanced HNSCC [87], and locally advanced CRC [88, 89], potentially suggesting that, at least in some patient populations, neoadjuvant ICIs may not require ICD induction to enable major clinical responses, a possibility that remains to be investigated in the TNBC setting.

In summary, combinatorial regimens involving one or more ICD inducer(s) and ICI-based immunotherapy appear to be superior to ICD inducers alone in a variety of clinical scenarios, but whether this truly reflects a positive therapeutic interaction rather than a pronounced efficacy of (at least some) ICIs employed as monotherapy generally remains to be formally investigated in patients.

## Unsuccessful interaction between ICD inducers and immunotherapy with ICIs

Not all preclinical and clinical studies completed thus far have documented a successful interaction between ICD induction by conventional chemotherapeutics, RT or targeted anticancer agents and ICI-based immunotherapy, calling for an improved understanding of the underlying (immuno)biological reasons for the design of optimized combinatorial regimens for clinical use.

### Preclinical evidence

Even in preclinical tumor models, ICD inducers and ICIs do not necessarily exhibit cooperative effects, at least in some cases owing to inappropriate dosing or administration schedules that promote the intratumoral or systemic accumulation of immunosuppressive cells.

In contrast to E0771 mammary carcinomas [46], MC38 CRCs established in immunocompetent C57BL/6 mice do not exhibit superior responses to the simultaneous administration of paclitaxel (20 mg/kg) and docetaxel (10 mg/kg) in combination with a PD-1 blocker, a lack of therapeutic interaction that appears to be accompanied by scarce recruitment of tumor-infiltrating lymphocytes to the tumor bed [90]. The concurrent administration of oxaliplatin (~10 mg/kg) plus a PD-L1 blocker to mice bearing subcutaneous CT26 CRCs also failed to significantly affect tumor growth and did not improve the intratumoral or systemic ratio between CD8^+^ T cells and immunosuppressive CD4^+^CD25^+^FOXP3^+^ regulatory T (T_REG_) cells [28, 91], indicating that additional immunostimulatory molecules, such as TLR7 agonists, may be required to enable this therapeutic interaction [26]. Moreover, the efficacy of both carboplatin (100 mg/kg) and paclitaxel (10 mg/kg) against mouse ID8 ovarian cancers established intraperitoneally has been shown to remain unaltered by the concomitant administration of an ICI targeting hepatitis A virus cellular receptor 2 (HAVCR2, also known as TIM-3) [92]. At least hypothetically, the inability of taxanes and oxaliplatin to cooperate with ICIs in immunocompetent mice bearing CRCs or ovarian cancers may reflect tumor-intrinsic features that prevent the optimal induction of ICD in vivo. This possibility, however, remains to be formally investigated.

Interestingly, in the latter experimental setting, employing a sequential (rather than concurrent) delivery schedule resulted in inferior efficacy coupled with the intraperitoneal accumulation of T_REG_ cells [92]. Similarly, while delivering cyclophosphamide (100 mg/kg) upfront reportedly synergizes with a CTLA4 blocker in CT26-bearing mice, inverting the order of administration appears to compromise therapeutic interactions along with an increase in CD8^+^ T-cell apoptosis [45, 93]. Notably, the same combinatorial regimen does not exhibit any efficacy in mice bearing subcutaneous RENCA renal cell carcinomas, which is at least correlated with the accumulation of myeloid-derived suppressor cells (MDSCs) and the consequent release of immunosuppressive cytokines [45, 94].

At least in some preclinical tumor models, including subcutaneous MC38 CRCs and H22 hepatocellular carcinomas growing in immunocompetent hosts, cisplatin also appears unable to unlock full-blown therapeutic responses to PD-1 blockers, irrespective of relative administration schedule [27, 32], which may reflect the limited ability of cisplatin to elicit ICD in some settings [37]. Comparable results have been obtained with in vivo syngeneic mesothelioma models treated with vinorelbine or 5-fluorouracil and dual PD-1/CTLA4 blockade, with AB1 (but not AE17) mesothelioma revealing some degree of antagonism [40]. Similarly, paclitaxel delivered intraperitoneally at the MTD (50 mg/kg) reportedly fails to cooperate with a PD-L1 blocker in immunocompetent C57BL/6 mice bearing EO771 TNBCs [95]. While paclitaxel has been shown to unlock the therapeutic efficacy of a PD-1 blocker in the same model [46], whether this apparent discrepancy reflects intrinsic immunobiological differences between PD-L1 and PD-1 signaling remains to be fully elucidated.

Collectively, these preclinical studies highlight the complexity of optimizing combinatorial regimens involving one or more ICD inducer(s) and ICIs, as an inappropriate dose or administration schedule can not only prevent these agents from cooperating but also (at least in some settings) can generate therapeutic antagonism, often in the context of local or systemic immunosuppression.

### Clinical evidence

Despite considerable expectations, numerous randomized phase II or III clinical trials have failed to demonstrate an advantage from combining ICIs with SOC ICD-inducing therapeutic regimens (Table 2).

**Table 2:** Unsuccessful clinical trials testing ICD inducers and ICIs^a^

Indication	ICD inducer(s)	ICI(s)	Other	Trial Name	Trial #	Ref.
Breast cancer	Cyclophosphamide	Tecemotide	Endocrine therapy	STRIDE	NCT00925548	n.a.
Breast cancer	Cyclophosphamide, doxorubicin, paclitaxel	Atezolizumab	Trastuzumab, pertuzumab	IMpassion050	NCT03726879	[118]
Colorectal carcinoma	5-fluorouracil, leucovorin, oxaliplatin	Nivolumab	Bevacizumab	CheckMate 9×8	NCT03414983	[105, 106]
Glioblastoma	RT, temozolomide	Ipilimumab, nivolumab	n.a.	n.a.	NCT04396860	[185]
Glioblastoma	RT, temozolomide	Nivolumab	n.a.	CheckMate 498	NCT02617589	[108]
Glioblastoma	RT, temozolomide	Nivolumab	n.a.	CheckMate 548	NCT02667587	[107]
HNSCC	Cetuximab, RT	Durvalumab	n.a.	n.a.	NCT03258554	[186]
HNSCC	AFX, cisplatin, SFX	Pembrolizumab	n.a.	KEYNOTE-412	NCT03040999	[187]
NSCLC	Carboplatin, cisplatin, gemcitabine, paclitaxel, pemetrexed	Epacadostat, nivolumab	n.a.	n.a.	NCT03348904	n.a.
NSCLC	Carboplatin, cisplatin, pemetrexed	Atezolizumab	n.a.	IMpower 132	NCT02657434	[97]
NSCLC	Carboplatin, paclitaxel	Ipilimumab	n.a.	n.a.	NCT02279732	n.a.
NSCLC	Carboplatin, paclitaxel	Ipilimumab	n.a.	n.a.	NCT01285609	[99]
NSCLC	Carboplatin, nab-paclitaxel, paclitaxel	Atezolizumab	n.a.	IMpower131	NCT02367794	[96]
Ovarian carcinoma	Carboplatin, paclitaxel	Atezolizumab	Bevacizumab	IMagyn050	NCT03038100	[119, 188]
Ovarian carcinoma	Carboplatin, paclitaxel	Avelumab	n.a.	JAVELIN Ovarian 100	NCT02718417	[189]
Ovarian carcinoma	Doxorubicin	Avelumab	n.a.	JAVELIN Ovarian 200	NCT02580058	[190]
Prostate cancer	Docetaxel	Pembrolizumab	Prednisone, dexamethasone	KEYNOTE-921	NCT03834506 NCT04907227	[109]
Prostate cancer	Docetaxel, RT	Ipilimumab	n.a.	n.a.	NCT00861614	[110]
SCLC	Etoposide, carboplatin, cisplatin	Ipilimumab	n.a.	n.a.	NCT01450761	[100]
SCLC	Platinum-based chemotherapy	Ipilimumab, nivolumab	n.a.	CheckMate 451	NCT02538666	[101]
SCLC	Platinum-based chemotherapy	Nivolumab	n.a.	CheckMate 331	NCT02481830	[102]
TNBC	Carboplatin, nab-paclitaxel	Atezolizumab	Surgery	NeoTRIPaPDL1	NCT02620280	[117]
TNBC	Paclitaxel	Atezolizumab	n.a.	IMpassion131	NCT03125902	[70]
Urothelial carcinoma	Carboplatin, cisplatin, gemcitabine	Pembrolizumab	n.a.	KEYNOTE-361	NCT02853305	[111]
Urothelial carcinoma	Carboplatin, cisplatin, gemcitabine	Atezolizumab	n.a.	IMvigor130	NCT02807636	[112, 191]

*AFX* accelerated fractionation radiotherapy, *HNSCC* head and neck squamous cell carcinoma, *ICD* immunogenic cell death inducer, *ICI* immune checkpoint inhibitor, *n.a.* not applicable, *NSCLC* non-small cell lung carcinoma, *RT* radiation therapy, *SCLC* small cell lung carcinoma, *SFX* standard fractionation radiotherapy, *TNBC* triple-negative breast cancer

^a^Limited to Phase II or higher; last updated: 10/17/2024

The IMpower131 and IMpower132 trials explored the addition of atezolizumab to SOC chemotherapeutic regimens with ICD-inducing activity in patients with NSCLC, highlighting potential benefits but also inconsistent improvements in disease outcome [96–98]. IMpower131 enrolled patients with advanced squamous NSCLC and compared the efficacy of atezolizumab vs placebo combined with carboplatin and either paclitaxel or nab-paclitaxel. While PFS was significantly improved in the atezolizumab arm, particularly in patients with PD-L1^+^ lesions, there was no significant extension in OS [96]. Virtually identical findings emerged from IMpower132, which randomly allocated patients with advanced nonsquamous NSCLC to atezolizumab *vs* placebo plus platinum-based chemotherapy (carboplatin or cisplatin plus pemetrexed) [97]. Moreover, the addition of the CTLA4 blocker ipilimumab to paclitaxel- and carboplatin-based chemotherapy failed to improve disease outcomes in two randomized, phase III clinical trials enrolling patients with squamous NSCLC (NCT01285609, NCT02279732) while significantly increasing the incidence of adverse events [99]. The large-scale CA184-156 trial tested the combination of etoposide and platinum-based chemotherapy optionally in combination with ipilimumab (followed by ipilimumab maintenance) in patients with extensive small lung cell carcinoma (SCLC), with outcomes that did not differ across study arms [100]. Similar findings were documented by the CheckMate 451 and CheckMate 331 studies, two randomized, phase III trials that enrolled patients with SCLC receiving first-line platinum-based chemotherapy (not earlier than 3 weeks later) with nivolumab, ipilimumab or placebo (CheckMate 451) [101], or with SCLC relapsing after platinum-based chemotherapy treated with nivolumab, topotecan, or amrubicin (CheckMate 331) [102]. Indeed, despite the lack of a placebo arm in the latter study, ICI-based immunotherapy delivered after ICD-inducing chemotherapy failed to enable clinical advantages, potentially owing to the delay between the two approaches.

Considerable challenges have also been documented in gastroesophageal and colorectal settings. For example, while the phase III KEYNOTE-062 trial, which investigated the combination of pembrolizumab plus chemotherapy (cisplatin plus 5-fluorouracil or capecitabine) in previously untreated patients with PD-L1^+^ advanced gastric or gastroesophageal junction adenocarcinoma, demonstrated a higher ORR in patients allocated to the combination treatment arm, it failed to result in improved PFS, OS, or response duration (but increased the rate and severity of side effects) [103, 104]. Moreover, the randomized phase II/III CheckMate 9×8 trial, which explored the addition of nivolumab to SOC FOLFOX plus bevacizumab-based chemotherapy in previously untreated patients with unresectable CRC, demonstrated a trend toward an improved 1-year PFS rate, ORR and response duration for the combinatorial regimen over SOC chemotherapy only but failed to meet its primary endpoint of PFS extension [105, 106]. Nivolumab not only failed to ameliorate disease outcomes in patients with newly diagnosed glioblastoma with a methylated or undetermined *MGMT* promoter subjected to surgery plus adjuvant RT plus temozolomide-based chemotherapy in the context of a randomized phase III CheckMate 548 clinical trial [107] but also failed to outperform temozolomide as a partner for adjuvant RT in patients with newly diagnosed glioblastoma with an unmethylated *MGMT* promoter in the context of the randomized phase III CheckMate 498 study [108]. Finally, the phase III KEYNOTE-921 trial demonstrated that the therapeutic activity of docetaxel in patients with metastatic castration-resistant prostate cancer (CRPC) cannot be ameliorated by the coadministration of pembrolizumab or ipilimumab [109], as did the randomized CA184-043 study, which randomly allocated men with metastatic CRPC who failed docetaxel-based chemotherapy and received bone-targeting RT to ipilimumab or placebo [110].

Trials investigating ICD-chemotherapeutics and ICIs in patients with urothelial carcinoma have faced similar obstacles. Neither pembrolizumab nor atezolizumab were able to improve OS extension afforded by carboplatin- or cisplatin-based SOC chemotherapy (in one study, optionally combined with gemcitabine) in patients with urothelial carcinoma enrolled in the randomized, phase III KEYNOTE-361 [111] and IMvigor130 [112] clinical studies, despite at least some PFS benefit associated with the administration of atezolizumab plus gemcitabine and platinum-based (especially cisplatin-based) chemotherapy [112]. Similarly, two randomized clinical trials investigating avelumab as a therapeutic partner for SOC chemoradiation in patients with locally advanced HNSCC (JAVELIN 100 and GORTEC-REACH) failed to document an OR benefit for the combinatorial regimens over SOC chemoradiation alone [113, 114]. Moreover, IMpassion131, a randomized phase III clinical study testing paclitaxel with atezolizumab or placebo in patients with TNBC, failed to demonstrate any improvement in PFS or OS for the combinatorial regimen over SOC chemotherapy, although these patients also received dexamethasone, which is a powerful immunosuppressant often required to limit adverse events (be they elicited by natural disease progression or treatment) in patients [115, 116], before at least the first two infusions of paclitaxel [70]. Similarly, neoadjuvant carboplatin plus nab-paclitaxel and atezolizumab, followed by an adjuvant anthracycline regimen, was not more efficient than the same regimen without atezolizumab and did not ameliorate pathological complete response among TNBC patients enrolled in the NeoTRIP Michelangelo randomized trial [117]. Finally, patients with high-risk, early HER2^+^ breast cancer receiving neoadjuvant atezolizumab in combination with dose-dense doxorubicin plus cyclophosphamide, followed by paclitaxel, trastuzumab, and pertuzumab enrolled in the Phase III IMpassion050 trial failed to experience pathological complete responses at increased rates compared with similarly treated women who received placebo instead of atezolizumab, neither in the intention-to-treat population nor among subjects with PD-L1^+^ tumors, ultimately leading to study discontinuation because of an unfavorable risk‒benefit ratio [118]. Similar dismal findings have been obtained by the randomized phase III IMagyn050 trial, which compared the efficacy of neoadjuvant atezolizumab *vs* placebo plus paclitaxel, carboplatin, and bevacizumab, followed by adjuvant bevacizumab, in patients with stage III/IV ovarian carcinoma [119].

Collectively, these observations suggest that ICD-inducing chemotherapeutic and radiotherapeutic regimens do not always cooperate with ICIs in the clinic, highlighting a critical need to understand the obstacles that currently limit the translation of existing preclinical data toward the development of combinatorial regimens with superior efficacy in patients, at least partially through changes in dose and administration schedule.

## Concluding remarks

**Fig. 2:**
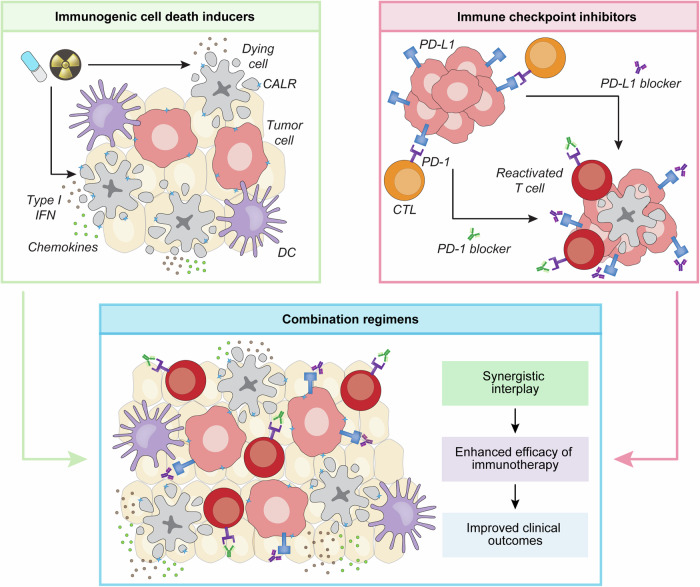
Potential synergy between ICD inducers and ICIs. Owing to their ability to recruit immune effector cells, including dendritic cells (DCs) and cytotoxic T lymphocytes (CTLs), immunogenic cell death (ICD)-inducing regimens (at least in some settings) can convert immunologically cold tumors into inflamed lesions. In this context, immune checkpoint inhibitors (ICIs), such as monoclonal antibodies targeting programmed cell death 1 (PDCD1, best known as PD-1) or CD274 (best known as PD-L1), which normally operate by (re)activating CTLs, may exhibit superior efficacy, providing a solid rationale for developing combinatorial clinical strategies potentially associated with improved clinical outcomes; CALR calreticulin, IFN interferon

In summary, an expanding preclinical literature suggests that various ICD-inducing cancer therapeutics can positively interact with ICIs across a panel of malignancies (Fig. 2). That said, such a positive interaction may not necessarily emerge from ICD induction but may rather reflect ICD-unrelated immunostimulatory effects that may support ICI sensitivity, as in vivo ICD induction remains challenging to assess [11, 120]. Moreover, despite such abundant preclinical findings, ICIs ameliorated the clinical efficacy of ICD-inducing anticancer agents delivered according to SOC dose and administration schedules in only a few clinical scenarios.

**Fig. 3:**
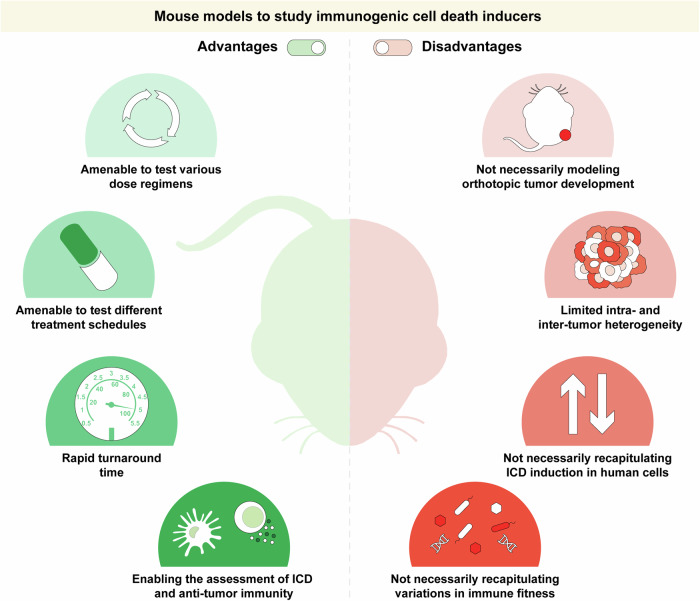
Advantages and limitations of current mouse models for the study of ICD. Most mouse models currently employed to investigate immunogenic cell death (ICD) induction are amenable to testing multiple (including noncanonical) dose regimens and administration schedules, offer rapid turnaround times and are compatible with the formal assessment of tumor-targeting immune responses. However, these models do not necessarily recapitulate human oncogenesis in terms of disease site or inter- and intratumor heterogeneity. Moreover, whether the murine system can properly model ICD induction in human cancer cells as well as whether systemic factors may influence immune fitness in patients with cancer remains to be formally established

Several factors may explain (at least in part) such a discrepancy between preclinical and clinical settings (Fig. 3). First, while in preclinical settings, ICD-inducing therapeutics can be easily delivered according to nonconventional doses, including metronomic schedules that have been consistently associated with increased immunogenicity [121–124], clinical trials combining ICD inducers and ICIs generally rely on SOC approaches, which have often been developed according to the MTD principle and hence may be associated with nonnegligible lympho- and myelosuppression [125–129]. In this context, it would be fundamental to test ICIs plus ICD inducers administered according to metronomic schedules or at doses lower than the MTD in clinical settings that may be compatible with such an approach, for example, in patients experiencing severe adverse events when ICD-inducing agents are delivered as per SOC. Second, most preclinical studies testing ICD inducers plus ICIs harness mouse cancer cell lines to establish subcutaneous tumors in syngeneic immunocompetent hosts, which (1) largely fail to recapitulate the intra- and interpatient heterogeneity of human tumors [130], and (2) offer to dying cancer cells a privileged and most often nonphysiological immunological contexture to elicit anticancer immunity [131, 132]. The use of mouse tumor models that develop orthotopically in the context of failing immunosurveillance, such as carcinogen-elicited or genetically driven neoplasms, may circumvent (at least in part) these limitations [133–135].

Third, most often, clinical trial design fails to build on preclinical data comparing different administration schedules for combining ICD inducers with ICIs (e.g., concurrent *vs*. sequential with ICIs first-in *vs*. sequential with ICD induction first-in) to achieve superior efficacy, which tends to exhibit at least some context dependency [21, 136]. A systematic preclinical assessment of administration schedules in immunocompetent tumor models is expected to assist in the identification of optimal regimens to combine ICD inducers with ICIs for translation to clinical testing, potentially reducing the number of trials ultimately reporting a lack of interaction between these treatment modalities. Fourth, ICD induction by chemotherapy, RT or targeted anticancer agents as formally assessable only in syngeneic mouse tumor models [137] may not necessarily result in similar efficacy in fully human systems (and notably cancer patients), potentially calling for the development of combinatorial ICD-inducing strategies. As a standalone example, cisplatin is a poor ICD inducer but may be converted into a powerful inducer by combining it with an endoplasmic reticulum stressor [37]. Fifth, while ICD induction in vitro is fairly straightforward, human tumors evolve as they establish numerous, not necessarily overlapping, mechanisms that limit the induction of ICD and its perception as immunogenic by the host [138–140]. Identifying these mechanisms, which may vary not only across tumor types but also across different malignant lesions in the same patient or even across different areas of the same tumor, on an individual basis may offer actionable mechanistic insights to develop superior combinations of ICD inducers and ICIs. Sixth, a number of variables affecting patient immune fitness may prevent ICD inducers from actually eliciting an ICI-active immune response, including (1) polymorphisms in genes encoding critical immune receptors [141], (2) alterations in the gut or intratumoral microbiome [142], (3) dietary habits [143], (4) comorbidities [144], and (5) medications and over-the-counter drugs [115, 145, 146]. Upon precise identification, many of these barriers may offer a means to (1) select patients at increased likelihood to benefit from therapeutic regimens involving ICD-inducing agents and ICIs and/or (2) improve the efficacy of such combinatorial strategies.

Finally, cancer cells exhibit extraordinary heterogeneity, not only across tumor types or in different patients with the same neoplasms but also across different tumors in the same patient and even within individual lesions [130]. This implies that specific therapeutics may elicit ICD in some but not all cancer cells, at least in part reflecting the high interconnectivity that characterizes cell death signaling modules, which ultimately impacts immunogenicity [147–149]. While spatially resolved omics technologies may offer an improved characterization of the heterogeneity of malignant lesions with respect to transcriptional, proteomic and metabolomic features [150], whether any of these parameters or combinations thereof may accurately predict the propensity of an individual tumor to respond to ICD inducers alone or combined with ICIs has yet to be demonstrated. Similarly, while a number of circulating factors are being scrutinized for their prognostic and predictive value in different oncological indications [151], whether these biomarkers can be used to efficiently identify patients with cancer at an increased likelihood of benefiting from ICD-inducing therapeutics in combination with ICIs remains unclear. As an added layer of complexity, a surge in the circulating levels of ICD-associated biomarkers such as high mobility group box 1 (HMGB1), which has been correlated with improved disease outcome in patients with breast carcinoma or HNSCC receiving ICD-inducing agents [152, 153], may *de facto* originate from ICD-unrelated processes, hence potentially being poorly predictive of a positive interaction with ICIs.

As such, outstanding challenges for the field include (but are not limited to): (1) the identification of new chemical entities or physical agents with superior ICD-inducing capacity that can be moved forward to clinical translation; (2) the characterization of novel, clinically relevant dosing schedules to increase the ICD-inducing potential of anticancer therapeutics commonly used in the clinic at (or close to) the MTD; (3) the deconvolution of novel cellular pathways leading to bona fide ICD; (4) the identification of preclinical tumor models that recapitulate the cancer‒immunity interaction as closely as possible to their human counterparts; (5) the use of such models toward an unbiased assessment of optimal combinatorial regimens with respect to the administration schedule; (6) the development of strategies that circumvent the natural tendency of human tumors to evade immunosurveillance; and (7) the identification of host of cancer-related factors that limit the perception of cell death as immunogenic and at the same time may be amenable to therapeutic targeting or aid patient stratification.

In conclusion, while additional preclinical and clinical work is needed to unlock the full therapeutic potential of ICD-inducing therapeutics as partners for ICIs, we surmise that addressing these obstacles, or at least taking them under attentive consideration as potential predictors of response during clinical trial design, may lead to the development of novel, safe and efficient combinatorial regimens for patients with a wide variety of malignancies.
